# Incorporating interactive workshops into bedside teaching: completion of a multi-modal rheumatology rotation significantly increases internal medicine residents’ competency and comfort with comprehensive knee examinations

**DOI:** 10.1186/s12909-022-03425-4

**Published:** 2022-05-10

**Authors:** Alysia Kwiatkowski, Najia Shakoor, Augustine Manadan, Joel A. Block, Sonali Khandelwal

**Affiliations:** 1 Division of Allergy, Immunology & Rheumatology, The State University of New York at Buffalo, Buffalo, NY USA; 2 Division of Rheumatology, Rush University Medical Center, Chicago, IL USA

**Keywords:** Workshop, Physical examination, Bedside teaching, Resident, Knee examination, Musculoskeletal

## Abstract

**Background:**

Studies have elucidated the lack of competency in musculoskeletal (MSK) examination skills amongst trainees. Various modalities have been studied, however, there remains a dearth of literature regarding the effectiveness of bedside teaching versus dedicated workshops. Our aim was to determine if incorporating a workshop into a rheumatology rotation would be effective in increasing medicine residents’ competency and comfort with knee examinations when compared to the rotation alone.

**Methods:**

Over 16 months, rotators were randomized to workshop plus rotation versus rotation alone. Participants were tested on their knee examination skills using an objective structured clinical examination (OSCE). Surveys were administered assessing to what degree the rotation was beneficial. Comfort and helpfulness were measured using a 5-point Likert scale. Paired and independent samples t-tests were used for comparisons.

**Results:**

Fifty-seven residents participated. For both groups, there were improvements between pre- and post-OSCE scores (workshop *p* < 0.001, no workshop *p* = 0.003), and levels of comfort with examination (workshop *p* < 0.001, no workshop *p* < 0.001). When comparing groups, there were differences favoring the workshop in post-OSCE score (*p* = < 0.001), mean change in OSCE score (*p* < 0.001) and mean change in comfort with knee examination (*p* = 0.025).

**Conclusion:**

An elective in rheumatology augmented residents’ MSK competency and comfort. Incorporation of a workshop further increased knowledge, skills and comfort with diagnosis and treatment. Current educational research focuses on alternatives to traditional methods. This study provides evidence that a multi-modal approach, combining traditional bedside and interactive models, is of benefit.

**Supplementary Information:**

The online version contains supplementary material available at 10.1186/s12909-022-03425-4.

## Background

Musculoskeletal (MSK) complaints are the most common conditions evaluated in the ambulatory care setting [1]. Though the prevalence of MSK pathology in the population is considerable, especially among the aging population [2], less than 3% of time in Liaison Committee on Medical Education accredited medical school curricula is devoted to MSK medicine [3]. As a result, graduating medical students have a considerable deficit in MSK knowledge [3, 4] that persists into post-graduate training and beyond.

In one study evaluating knowledge, 210 graduating medical students in the United Kingdom were given a validated assessment of MSK knowledge [5]. Only 21% of students passed the examination and 40% rated themselves as competent in MSK medicine. In another study [6], 170 postgraduate and faculty participants took a practical test of anatomic structures commonly involved in rheumatic diseases. When the entire cohort was considered, the mean correct answer was 46.6% with rheumatology fellows scoring significantly higher than non-rheumatologists.

Various teaching modalities have been developed to attempt to remedy known gaps including lecture-based [7–9], peer-to-peer [10–13] and workshop-based learning [14–18]. Though a surplus of high-quality evidence is lacking [19], much of the previously published literature supports the use of patient educators, small group sessions, computer assisted learning and especially workshops in the enhancement and retention of MSK knowledge and skills.

In an Irish study [20], 140 fourthyear medical students rotated through a new interactive musculoskeletal module over the course of 2 weeks. This module included lectures, interactive tutorials, case discussions, and clinical examination demonstrations. Students found the interactive tutorial approach (48%) to be the most effective teaching method.

Inter and multidisciplinary teaching has been shown to be effective in increasing knowledge gaps [21, 22]. In one study [21], a “musculoskeletal week” program was developed and presented to internal medicine, physical medicine and rehabilitation, and orthopedic residents as well as students and residents from other health profession programs. Faculty from multiple specialties taught skills with practical application. Self-reported scores and confidence with shoulder and knee complaints increased significantly and these were confirmed by evaluation.

Traditional teaching (i.e., at the bedside) has been the mainstay of both undergraduate and graduate medical education. Subspecialty elective rotations have been shown to increase specific skills and knowledge [23]. In a study by Goldenberg et al. [24], 24 internal medicine residents participated in a rheumatology elective (12 ambulatory and 12 inpatient). Both groups performed significantly better in tests of knowledge and clinical performance compared to groups of residents and medical students that did not undergo the elective.

To our knowledge, no studies have been performed comparing the effectiveness of traditional bedside teaching alone versus the addition of dedicated workshops. The aim of this study was to determine if incorporating a MSK workshop into a 2-week clinical rotation in rheumatology would result in an increase internal medicine residents’ competency and comfort with knee examinations when compared to the rotation alone.

## Methods

### Ethical approval and sample recruitment

**Fig. 1:**
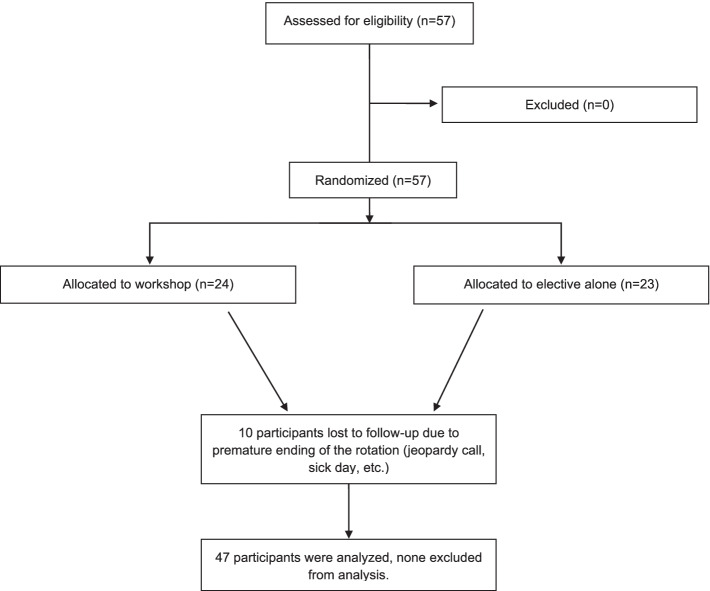
Participant enrollment flow diagram

The Rush University Medical Center Institutional Review Board granted approval for the study and informed consent was obtained from all participants. This was a single center study. Internal medicine and preliminary residents of all PGY levels were asked to voluntarily participate at the start of the elective. No resident declined to participate. The rheumatology elective curriculum is compromised equally of inpatient and outpatient experiences over a 2-week period, without formal didactics on musculoskeletal examination skills. During the 16-month study period, from January 2018 to April 2019, each block of resident rotators were randomized to workshop plus rotation versus rotation alone, with every other group receiving the workshop (Fig. 1). There were no exclusion criteria.

### Pre-rotation evaluation

Participants were tested on their knee examination skills at the start of the rotation by one of two trained evaluators using an objective structured clinical examination (OSCE), graded on a linear scale, for a total of 14 points. For every maneuver completed correctly, 1 point was given. For an element attempted but completed incorrectly, 0.5 points was given (Additional file 1: Appendix A).

### Workshop design

Those randomized to the intervention group were provided a 1-hour workshop, which was designed with input from a rheumatology faculty focus group. The session consisted of a didactic presentation (based on *Bates’ Guide to Physical Examination and History Taking* [25]), video (The New England Journal of Medicine, Videos in Clinical Medicine- *Clinical Evaluation of the Knee* [26]) and supervised hands-on application of skills. For the didactic portion, a PowerPoint© was used which consisted of a review of anatomical structures and physical examination techniques (inspection, palpation, assessing for fluid, range of motion testing, and provocative tests). For the skills component, residents were partnered and practiced the physical examination on each other with direct attending oversight and demonstration. Immediately following the rotation, all residents were retested using the OSCE.

### Pre- and post-rotation evaluation

Residents were administered a pre- and post-rotation survey assessing to what degree the rotation enhanced their comfort with knee examination skills, comfort with diagnosing and treating common musculoskeletal complaints, and to what degree elective completion was helpful. Participants were also asked to attribute what percentage of various experiences contributed to their total musculoskeletal knowledge (totaling 100%). Examples of these experiences included skills learned during medical school, completion of a rheumatology elective, and independent study.

### Statistical analysis

Comfort and helpfulness were measured using a 5-point Likert scale (1: not comfortable and 5: very comfortable). Paired and independent samples t-tests were used for pre and post as well as between group comparisons. Inter-rater variability was measured between the two OSCE evaluators. The significance value alpha was set to 0.05 for these analyses. Statistical analysis was completed using SPSS software, version 22.

## Results

### Cohort and Interrater variability

Fifty-seven residents participated in the study; ten were lost to follow-up due to being removed from the rotation for call obligations, or taking a sick day. As the number of residents assigned to rheumatology varied biweekly, group sizes were unequal with 24 receiving the workshop and 23 completing the elective alone. Only 10% were aware of this study prior to rotation start. Baseline characteristics pre-rotation are shown in Table 1. There were no statistically significant differences between the workshop and no workshop groups at baseline. Inter-rater variability was calculated using the intraclass correlation coefficient and was found to be 0.995–0.997. This is considered to be excellent [27] per Koo and Li, 2016.

**Table 1:** Baseline Characteristics (Mean ± Standard Deviation)

	Workshop	No Workshop	Sig
Pre-Rotation OSCE	5.4 ± 2.0	6.5 ± 2.7	0.107
Pre-Rotation Comfort with Knee Examination	2.7 ± 0.9	3.2 ± 1.0	0.098
Pre-Rotation Number of Comfortable Diagnoses	1.7 ± 1.3	1.9 ± 1.2	0.066
Pre-Rotation Number of Comfortable Treatments	1.5 ± 1.1	1.6 ± 1.3	0.344
Pre-Rotation Medical School Contribution to MSK Knowledge	63.7 ± 28.7	57.0 ± 23.2	0.592
Pre-Rotation Rheum Elective Contribution to MSK Knowledge	7.8 ± 12.7	10.9 ± 17.8	0.642
Pre-Rotation Independent Study Contribution to MSK Knowledge	16.7 ± 20.5	17.5 ± 14.4	0.605

### Intravariable analysis-experimental group

As shown in Table 2, for the workshop group, there were significant improvements between pre- and post-OSCE scores (*p* < 0.001), levels of comfort with knee examination skills (*p* < 0.001), the number of lower extremity MSK conditions that residents felt comfortable diagnosing (*p* < 0.001), and treating (*p* = 0.003). Of the experiences that residents felt contributed to their musculoskeletal knowledge, only completion of the rheumatology elective showed significant contribution (*p* < 0.001).

**Table 2:** Pre-Rotation Versus Post-Rotation Outcomes (Mean ± Standard Deviation)

	Workshop			No Workshop		
	Pre-Rotation	Post-Rotation	Sig	Pre-Rotation	Post-Rotation	Sig
OSCE Score	5.4 ± 2.0	11.8 ± 1.2	**< 0.001**	6.5 ± 2.7	8.7 ± 2.8	**0.003**
Comfort with Knee Examination	2.7 ± 0.9	4.3 ± 0.7	**< 0.001**	3.2 ± 1.0	4.0 ± 0.5	**< 0.001**
Number of Comfortable Diagnoses	1.7 ± 1.3	3.1 ± 1.4	**< 0.001**	1.9 ± 1.2	2.2 ± 1.5	0.296
Number of Comfortable Treatments	1.5 ± 1.2	2.6 ± 1.5	**0.003**	1.6 ± 1.0	2.0 ± 1.3	0.131
Rheumatology Elective Contribution to MSK Knowledge	7.8 ± 12.7	33.3 ± 17.0	**< 0.001**	10.9 ± 17.8	34.9 ± 20.7	**< 0.001**

### Intravariable analysis-control group

For the no workshop group, also shown in Table 2, there were significant improvements between pre- and post-test OSCE scores (*p* = 0.003) and levels of comfort with knee examination skills (*p* < 0.001), and the percentage of total MSK knowledge that rheumatology elective completion resulted in (*p* < 0.001). No significant differences were found between the number of lower extremity MSK conditions that residents felt comfortable diagnosing (*p* = 0.296), and treating (*p* = 0.131).

### Post-rotation group comparative analysis

When comparing groups post-rotation, as shown in Table 3, there were significant differences favoring the workshop group in post-OSCE score (*p* = < 0.001), helpfulness of the rotation in enhancing MSK examination skills (*p* = 0.033) and the number of lower extremity MSK conditions that residents felt comfortable diagnosing (*p* = 0.046). There were no significant differences post-rotation between the control and experimental group in the number of lower extremity MSK conditions that residents felt comfortable treating. Similarly, there was no difference noted in total MSK knowledge from the rheumatology elective.

**Table 3:** Post-Rotation Variables (Mean ± Standard Deviation)

	Workshop	No Workshop	Sig
Post-Rotation OSCE	11.8 ± 1.2	8.7 ± 2.8	**< 0.001**
Post-Rotation Comfort with Knee Examination	4.3 ± 0.7	4.1 ± 0.5	0.219
Post-Rotation Number of Comfortable Diagnoses	3.1 ± 1.4	2.2 ± 1.5	**0.046**
Post-Rotation Number of Comfortable Treatments	2.6 ± 1.5	2.0 ± 1.3	0.136
Post-Rotation Rheum Elective Contribution to MSK Knowledge	33.3 ± 17.0	34.9 ± 20.7	0.739
Helpfulness of Rotation in Enhancing Exam Skills	4.7 ± 0.7	4.3 ± 0.6	**0.033**
Helpfulness of Rotation in Diagnosing MSK conditions	4.2 ± 0.9	4.0 ± 0.7	0.401
Helpfulness of Rotation in Treating MSK conditions	4.2 ± 0.9	4.0 ± 0.7	0.369

### Group comparative analysis-outcomes

When comparing the workshop to the no workshop group, there were significant differences favoring the workshop group in mean change in OSCE scores (mean absolute change workshop = 6.33 ± 2.06; mean absolute change no workshop = 2.30 ± 3.17, *p* < 0.001) and comfort with knee examinations (mean absolute change workshop = 1.57 ± 0.843; mean absolute change no workshop = 0.96 ± 0.93, *p* = 0.025) pre- and post-rotation.

## Discussion

To our knowledge, this is the only study directly comparing subspecialty elective completion to an elective combined with a MSK workshop. Existing literature supports a lack of competency in application of MSK examination skills [28–31]. In a study by Schmale [28], all second through fourth- year medical students were invited to participate in a survey consisting of short answer questions on MSK medicine previously validated for MSK competency. Results revealed increasing scores by level of education. Percent passing ranged from 0% for the second-year students to 43% at the fourth-year level. Shortfalls in MSK knowledge persisted post-medical school graduation. In another study [29], 300 primary care physicians were selected and completed a questionnaire and an assessment of cognitive competency in rheumatology. While the survey indicated that MSK complaints made up 30–40% of their practice, only 25% achieved a passing score. In concordance with the existing literature, we were able to demonstrate a baseline deficiency in MSK knee examination skills for internal medicine residents as evidenced by low baseline OSCE scores across the group as a whole.

Our results add further evidence that internal medicine residents also lack confidence in clinical skills as illustrated by low levels of baseline comfort with knee examinations and baseline perceived ability to diagnose and treat MSK conditions. In a study by Katz and Oswald [30, 32], 216 Canadian internal medicine residents received a survey ranking self-confidence in specialty skills. Self-confidence in rheumatology was the lowest of the specialties. In another study by Kroop et al. [31, 33], a self-assessed confidence survey was administered to PGY-1 and PGY-3 internal medicine residents. The survey assessed confidence in performing a rheumatologic history and physical examination, procedures, ordering/interpreting lab tests and caring for patients with rheumatologic conditions. Self-assessed confidence in joint procedures was consistently low in both groups. When comparing PGY 3 residents who did or did not take a rheumatology elective, the confidence was higher for exam skills and shoulder injection in residents who completed the elective. Among the 57 internal medicine residents who participated in our study, we were able to show that rheumatology elective completion alone significantly enhanced competency and comfort with musculoskeletal examination skills, aligning with this and with previously published literature on elective experiences [23, 24].

In a study by Hergenroeder et al. [16], 58 pediatric residents during a 1-month adolescent medicine rotation received a workshop on the knee and ankle. The workshop consisted of a video, direct observation, and demonstration of the technique by the resident. The residents increased their correct performance significantly and this effect was maintained at 9 months. In another study by Denizard-Thompson et al. [14], 36 residents received a half-day session on shoulder and knee complaints. This included a presentation highlighting history, examination and procedural skills and a charades game in which competitors demonstrated joint examinations. After the session, residents showed significantly increased confidence in MSK examinations and injections. When comparing those receiving a workshop in addition to a rheumatology elective to those who completed the elective alone, we also observed important differences. The workshop group scored significantly higher on the post-rotation OSCE, had greater increases in comfort with examination and had higher levels of perceived helpfulness of the rotation in both enhancing MSK skills and diagnosing MSK conditions. This supports the use of interactive workshops in increasing knowledge and clinical skills [14, 15].

Our study has several limitations. A power calculation was not performed and thus sample size adequacy could not be formulated. Ten residents were lost to follow-up, decreasing our possible sample size. Additionally, the study was conducted at a single tertiary academic center, perhaps limiting generalizability. Though we did not specifically analyze outcome by PGY level, we were able to demonstrate that the groups were similar at baseline in OSCE scores and levels of comfort. Another limitation includes evaluating lack of long-term retention rates of physical examination skills between the groups.

Future directions include replicating the study with more participants, pursuing multi-institutional collaboration, investigating outcomes within other specialties/sub-specialties and exploring long term retention rates.

## Conclusion

This study showed that an elective experience in rheumatology augmented internal medicine residents’ MSK competency and comfort. Incorporation of an interactive MSK workshop further increased residents’ knowledge, skills and comfort with diagnosing and treating rheumatologic conditions more than the elective alone. The current climate in educational research focuses on alternative approaches to traditional teaching methods. This study provides considerable evidence that a multi-modal approach in post-graduate education, combing traditional bedside and interactive models, is of benefit.

## Supplementary Information

**Additional file 1.**

**Additional file 2.**

**Additional file 3.**

## Data Availability

The datasets used and/or analyzed during the current study are available from the corresponding author on reasonable request.

## Abbreviations

MSK: Musculoskeletal
OSCE: Observed structure clinical examination
