# Supplementary material for: Incorporating interactive workshops into bedside teaching: completion of a multi-modal rheumatology rotation significantly increases internal medicine residents’ competency and comfort with comprehensive knee examinations
Source: BMC Med Educ. 2022 May 10;22:355. doi: 10.1186/s12909-022-03425-4 (PMC9092684; doi:10.1186/s12909-022-03425-4)
Supplement: Supplementary file 1 — Additional file 1. [file 12909_2022_3425_MOESM1_ESM.pdf]

Rush University Rheumatology

Objective Structured Clinical Examination

Resident Number: __________ Date: _____________

Task	Completed Correctly	Attempted Incorrectly	Did Not Attempt
**Inspection:**			
Gait			
Alignment			
**Palpation:**			
Tibiofemoral Joint			
Medial and Lateral Joint Compartments			
Patellofemoral Compartment			
Palpation for Effusion*			
**Range of Motion Testing:**			
Passive flexion, extension, internal rotation and external rotation			
Active flexion, extension, internal rotation and external rotation			
**Provocative Maneuvers:**			
Patellar Grind			
McMurray Test			
Adduction (or Valgus) Stress Test			
Adduction (or Varus) Stress Test			
Anterior Drawer Sign or Lachman Test			
Posterior Drawer Sign			
		**Total**	

* Bulge sign, balloon sign or balloting the patella are all acceptable.

A score of 1 will be given for all tasks completed correctly. A score of 0.5 will be given for all tasks attempted but not completely correctly. A score of zero will be given for items that were not attempted or were grossly incorrect.
