# Supplementary material for: Incorporating interactive workshops into bedside teaching: completion of a multi-modal rheumatology rotation significantly increases internal medicine residents’ competency and comfort with comprehensive knee examinations
Source: BMC Med Educ. 2022 May 10;22:355. doi: 10.1186/s12909-022-03425-4 (PMC9092684; doi:10.1186/s12909-022-03425-4)
Supplement: Supplementary file 2 — Additional file 2. [file 12909_2022_3425_MOESM2_ESM.docx]

Resident Number: __________ Date: _____________

1. Were you aware of this study prior to rotation start? Yes No
2. To what degree do you feel comfortable with performing an independent comprehensive knee examination?
   1. Very uncomfortable
   2. Somewhat uncomfortable
   3. Neither uncomfortable or comfortable (Neutral)
   4. Somewhat comfortable
   5. Very comfortable
3. To what degree do you feel completion of a rheumatology elective **was helpful** in enhancing your comfortability with performing an independent comprehensive knee examination?
   1. Very unhelpful
   2. Somewhat unhelpful
   3. Neither unhelpful or helpful (Neutral)
   4. Somewhat helpful
   5. Very helpful
4. To what degree do you feel comfortable with **recognizing and diagnosing** common rheumatologic conditions such as: rheumatoid arthritis, systemic lupus erythematosus, vasculitis, osteoarthritis, gout, calcium pyrophosphate deposition and fibromyalgia?

Which conditions do you feel the most comfortable diagnosing?

______________________________________

­­­­­­­­­­­­­______________________________________

- 1. Very uncomfortable
  2. Somewhat uncomfortable
  3. Neither uncomfortable or comfortable (Neutral)
  4. Somewhat comfortable
  5. Very comfortable

1. To what degree do you feel completion of a rheumatology elective **was helpful** in enhancing your comfortability with **recognizing and diagnosing** common rheumatologic conditions as listed in Question #4?
   1. Very unhelpful
   2. Somewhat unhelpful
   3. Neither unhelpful or helpful (Neutral)
   4. Somewhat helpful
   5. Very helpful
2. To what degree do you feel comfortable with **treating** common rheumatologic conditions such as: rheumatoid arthritis, systemic lupus erythematosus, vasculitis, osteoarthritis, gout, calcium pyrophosphate deposition and fibromyalgia?

Which conditions do you feel the most comfortable treating?

______________________________________

­­­­­­­­­­­­­______________________________________

- 1. Very uncomfortable
  2. Somewhat uncomfortable
  3. Neither uncomfortable or comfortable (Neutral)
  4. Somewhat comfortable
  5. Very comfortable

1. To what degree do you feel completion of a rheumatology elective **was helpful** in enhancing your comfortability with **treating** common rheumatologic conditions as listed in Question #6?
   1. Very unhelpful
   2. Somewhat unhelpful
   3. Neither unhelpful or helpful (Neutral)
   4. Somewhat helpful
   5. Very helpful
2. From the list below, please circle the conditions you feel most comfortable **diagnosing**.

Lumbar strain/sprain

Herniated disk

Spinal stenosis

Mechanical neck pain

Neurologic neck pain

Rotator cuff tendonitis

Rotator cuff tear

Bicipital tendonitis

Adhesive capsulitis

Acromioclavicular syndromes

Epicondylitis

Olecranon bursitis

Carpal Tunnel Syndrome

De Quervain tenosynovitis

Bursitis

Iliotibial band syndrome

Meniscal injury

Ankle sprain

Plantar Fasciitis

1. To what degree do you feel completion of a rheumatology elective **was helpful** in enhancing your comfortability with **recognizing and diagnosing** common musculoskeletal conditions as listed in Question #8?
   1. Very unhelpful
   2. Somewhat unhelpful
   3. Neither unhelpful or helpful (Neutral)
   4. Somewhat helpful
   5. Very helpful
2. From the list below, please circle the conditions you feel most comfortable **treating**.

Lumbar strain/sprain

Herniated disk

Spinal stenosis

Mechanical neck pain

Neurologic neck pain

Rotator cuff tendonitis

Rotator cuff tear

Bicipital tendonitis

Adhesive capsulitis

Acromioclavicular syndromes

Epicondylitis

Olecranon bursitis

Carpal Tunnel Syndrome

De Quervain tenosynovitis

Bursitis

Iliotibial band syndrome

Meniscal injury

Ankle sprain

Plantar Fasciitis

1. To what degree do you feel completion of a rheumatology elective was helpful in enhancing your comfortability with **treating** common musculoskeletal conditions as listed in Question #10?
   1. Very unhelpful
   2. Somewhat unhelpful
   3. Neither unhelpful or helpful (Neutral)
   4. Somewhat helpful
   5. Very helpful
2. How comfortable do you feel with your preparedness for the American Board of Internal Medicine Certifying Exam (i.e. Internal Medicine Board Examination) with regards to the Rheumatology and Musculoskeletal portions?
   1. Very uncomfortable
   2. Somewhat uncomfortable
   3. Neither uncomfortable or comfortable (Neutral)
   4. Somewhat comfortable
   5. Very comfortable
3. In the past two weeks, on how many days of the week do you read on rheumatology specific topics for 20 minutes or more?
   1. Zero days
   2. 1-2 days
   3. 3-4 days
   4. 5-6 days
   5. 7 or more days
4.
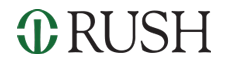
With a total of 100%, please indicate below the percentages each of the following have contributed to your musculoskeletal physical examination skills during residency:

| Skills learned during medical school: | % |
| --- | --- |
| Completion of a rheumatology elective: | % |
| Completion of an orthopedic surgery elective: | % |
| Completion of a sports medicine elective: | % |
| Independent study: | % |
| Other (please indicate): | % |
| Total | 100% |
